# How Strong Is the Evidence for Sodium Bicarbonate to Prevent Contrast-Induced Acute Kidney Injury After Coronary Angiography and Percutaneous Coronary Intervention?

**DOI:** 10.1097/MD.0000000000002715

**Published:** 2016-02-18

**Authors:** Yuhao Dong, Bin Zhang, Long Liang, Zhouyang Lian, Jing Liu, Changhong Liang, Shuixing Zhang

**Affiliations:** From the Department of Radiology, Guangdong Academy of Medical Sciences/Guangdong General Hospital, Guangzhou, Guangdong Province (YD, BZ, LL, ZL, JL, CL, SZ); Shantou University Medical College, Shantou (YD); and Graduate College, Southern Medical University, Guangzhou, China (BZ, LL).

## Abstract

Hydration with sodium bicarbonate is one of the strategies to prevent contrast-induced acute kidney injury (CI-AKI). The purpose of this study was to determine how strong is the evidence for sodium bicarbonate to prevent CI-AKI after coronary angiography (CAG) and/or percutaneous coronary intervention (PCI).

We conducted PubMed, EMBASE, and CENTRAL databases to search for randomized controlled trials (RCTs) comparing the efficacy of sodium bicarbonate with sodium chloride to prevent CI-AKI after CAG and/or PCI. Relative risk (RR), standardized mean difference (SMD), or weighted mean difference (WMD) with 95% confidence intervals (CIs) was calculated. Heterogeneity, publication bias, and study quality were evaluated, sensitivity analyses, cumulative analyses, and subgroup analyses were performed. The risk of random errors was assessed by trial sequential analysis (TSA).

Sixteen RCTs (3537 patients) met the eligibility criteria. Hydration with sodium bicarbonate showed significant beneficial effects in preventing CI-AKI (RR 0.67; 95% CI: 0.47–0.96, *P* = 0.029), decreasing the change in serum creatinine (SCr) (SMD −0.31 95% CI: −0.55 to −0.07, *P* = 0.011) and estimated glomerular filtration rate (eGFR) (SMD −0.17 95% CI: −0.30 to −0.04, *P* = 0.013). But no significant differences were observed in the requirement for dialysis (RR 1.11; 95% CI: 0.60–2.07, *P* = 0.729), mortality (RR 0.71; 95% CI: 0.41–1.21, *P* = 0.204) and reducing the length of hospital stay (LHS) (WMD −1.47; 95% CI: −4.14 to 1.20, *P* = 0.279). The result of TSA on incidence of CI-AKI showed the required information size (RIS = 6614) was not reached and cumulative z curve did not cross TSA boundary. The result of TSA on the requirement for dialysis and mortality demonstrated the required information sizes (RIS = 170,510 and 19,516, respectively) were not reached, and the cumulative z-curve did not cross any boundaries.

The evidence that sodium bicarbonate reduces the incidence of CI-AKI is encouraging but more well-designed randomized controlled trails are required to allow definitive firm conclusion to be drawn.

## Abstract 2

Supplemental Digital Content is available in the text

## INTRODUCTION

Coronary revascularization by percutaneous coronary intervention had been reported as an essential treatment for patients with stable artery disease. In the United States and Europe, the number of patients undergoing PCI reaches about 1.5 million every year. An important neglected complication of PCI is contrast-induced acute kidney injury (CI-AKI).^1,2^ The current oriented studies showed that the incidence of CI-AKI, which is defined as an increase in the serum creatinine (Scr) level of 25% or an increase of 0.5 mg/dL (or 44 μmol/L) from baseline within 48 to 72 hours of contrast exposure, ranges from 2.0% in those patients with normal baseline renal function to as high as 20% to 30% in those patients with a baseline creatinine >176 μmol/L (or 2.0 mg/dL) for patients undergoing coronary angiography (CAG) and/or PCI, which is far over that of the CT and other intravenous contrast injection procedures (less than 5% for outpatients).^1,3^ This could be due to the fact that the required dosage of contrast media is much larger in PCI and CAG. During the procedure, the contrast media should be injected discontinuously until the final diagnosis or treatment achieved. On the other hand, reduced effective circulatory volume resulted from the predispositional cardiac dysfunction and long operating time aggravated the kidney injury and making it more vulnerable to develop CI-AKI in patients undergoing CAG and/or PCI.^4^ Therefore, preparatory hydration is very essential in preventing CI-AKI in patients undergoing CAG and PCI.^5^ The 2012 American College of Cardiology Foundation/American Heart Association (ACCF/AHA) guidelines gave a class I (level B) recommendation for patients who are undergoing CAG or PCI to receive adequate preparatory hydration.^6^ Over the few decades, there had been quantities of randomized clinical trials and meta-analysis demonstrating the significant beneficial effects in sodium bicarbonate to prevent CI-AKI^7–19^ while there were also some demonstrating no consistent answer as to its efficacy.^20,21^ However, the way of trial conduction varied and random errors accumulated as the meta-analyses updated, which could have led to false-positive results.^22^

Our objective was to assess how strong is the evidence for sodium bicarbonate versus sodium chloride to prevent CI-AKI in patients undergoing CAG and/or PCI. Given small sample size of the published trials and the dangers of overestimating effect sizes with traditional meta-analysis, especially in prematurely terminated trials and for outcomes that were not powered for, our objective was also to use robust methodology trial sequential analysis (TSA) to critically evaluate whether the current accumulated data provide firm evidence to support sodium bicarbonate for prevention of CI-AKI and other relevant outcomes with randomized clinical trials that are of strictly accessed quality.

## METHODS

### Ethical Approval and Patient Consent

As this was a meta-analysis that did not involve identifiable patient data, no particular ethical considerations or patient consents were required.

### Literature Search

Literature search was conducted in the electronic databases PubMed, EMBASE, and Cochrane Central Register of Controlled Trials (CENTRAL) from their inception through August 3, 2015 to identify RCTs that reported comparison of effects of sodium bicarbonate versus sodium chloride on incidence of CI-AKI after CAG and/or PCI. Predefined search terms included “sodium bicarbonate,” “sodium chloride,” “saline,” “contrast-induced nephropathy,” “contrast-induced acute kidney injury,” “coronary angiography,” and “percutaneous coronary intervention.” No language restrictions were applied. The retrieved references of the included RCTs were also reviewed to determine additional studies not indexed in the common databases.

### Eligibility Criteria

Eligible trials should fulfill the following criteria: RCTs comparing hydration of sodium bicarbonate with sodium chloride in patients undergoing CAG and/or PCI. RCTs were defined as clinical trials in which individuals or other units were assigned to different treatment groups using randomization allocation, such as random number, computer-generated random sequences; if N-acetylcysteine (NAC) was infused, it was needed to be added in 2 arms; reporting data on the incidence of CI-AKI at least, the requirement of hemodialysis, mortality, length of hospital stay, levels of serum creatinine pre- and postprocedures, change in eGFR, and urine pH were not required as a must; and with high quality (Jadad score >2).

### Quality Assessment, Data Collection, and Clinical Outcomes

All trials were evaluated by 2 authors (BZ and YD) using the Jadad scale (5-point) to assess randomization (0–2 points), double blinding (0–2 points), and withdrawals or dropouts (0–1 point). Concealment of allocation was assessed in terms of adequate, inadequate, or unclear according to the Cochrane Handbook.^23^ A trial scoring more than 2 is deemed high quality. We extracted studies’ characteristics including study name, simple size, age, male (%), contrast media used, definition of CI-AKI, regiment, endpoints. Data were collected and arranged into a standardized form. Rigorous quality assessment was conducted, with the nonconcurrent or improperly constructed control groups prospectively excluded. Results were compared and discrepancies were resolved by further discussion. Clinical outcomes were arranged as primary and secondary endpoints. The primary endpoints were the development of CI-AKI, the requirement of dialysis, and mortality as defined in previous studies.^23^ The secondary endpoints included the duration of hospital stay, change in levels of serum creatinine eGFR, and urine pH pre- and postprocedures.

### Meta-Analysis and Heterogeneity Testing

The meta-analysis was performed in line with recommendation from the Cochrane collaboration and the Preferred Reporting Items for Systematic Reviews and Meta analyses (PRISMA) statement (Additional file 2), using STATA software, version 12.0 (Stata Corp LP, College Station, TX). All of the analyses were based on an “intention-to-treat” bias. Treatment effects of sodium bicarbonate were quantified by summary relative risk (RR) adopted from fixed or random effects model.^23,24^ Heterogeneity was assessed by Mantel–Haenszel method derived Cochran's Q statistic and associated I2 statistic, with values of 0% to 30%, 31% to 50%, and >50% representing mild, moderate, and substantial heterogeneity, respectively.

### Sensitivity Analysis

We performed sensitivity analysis using STATA software to assess the effect of an individual study on the pooled estimator by removing it in turn at a time. If the point estimate of the single study lays outside the 95% confidence interval (CI) of the pooled estimator, the study is suspected of contributing the most to heterogeneity.^23,25^

### Publication Bias and Regression Analysis

Evidence of publication bias was tested by funnel plot, Begg test,^26^ and Egger test.^25,27^ Standard meta-regressions of the effect size expressed as logRR were performed against trial-level covariates including publication year, sample size, contrast osmolality, with or without bolus, elective or urgent, and the definition of CI-AKI, to find out where the heterogeneity lied. All meta-regressions were weighted by the Dersimonian–Laird of each study.

### Subgroup Analyses

Subgroup analyses were performed to assess the effect of sodium bicarbonate versus sodium chloride in various conditions, including low osmolar versus iso-osmolar contrast agent, sample size >200 versus sample size <200, emergency versus elective procedures, and continuous versus bolus infusion of sodium bicarbonate.

### Cumulative Meta-Analyses

Cumulative analysis was applied to compare pooled RRs of all studies published chronologically to a specific point in time, aiming to figure out the tendency of summarized RRs according to the publication year before trial sequential analysis.

### Trial Sequential Analysis

Cumulative meta-analyses and repetitive testing of accumulating data run the risks of producing random errors. TSA is similar to interim analyses in a single trial, where monitoring boundaries are used to decide whether a trial could be terminated early when a *P* value is sufficiently small to show the anticipated effect or for futility.^28^ To minimize random errors, we performed the TSA analysis of the incidence of CI- AKI, the requirement of dialysis as well as the mortality using TSA program version 0.9 beta (www.ctu.dk/tsa), anticipating a certain relative risk reduction for efficacy outcome, α = 5%, 1 − β = 80% and estimating the diversity-adjusted required information size. The predefined relative risk reduction is 30% for the incidence of CI-AKI and mortality, but 11% relative risk increase for the requirement of dialysis. This relative risk reduction or increase was based on the results of previous meta-analysis^29^ or based on trials with low bias risk. Risk of bias assessment includes the following domains: sequence generation; concealment of allocation; blinding of participants and investigators; blinding of outcome assessors; completeness of outcome data; selective outcome reporting bias; and other bias. Risk of bias was classified as low, unclear, or high.^30^ For trial sequential analysis, results crossing the conventional boundary of significance (Z = ± 1.96, *P* < 0.05) but not the trial sequential monitoring boundary (TSMB) were defined as spuriously significant.^30^ Firm evidence was assumed to be reached when the Z-curve crossed the required information size (RIS) and the conventional boundary or crossed the TSMB before the RIS was reached.^31^

## RESULTS

### Literature Search Strategy and Included Trials

**FIGURE 1:**
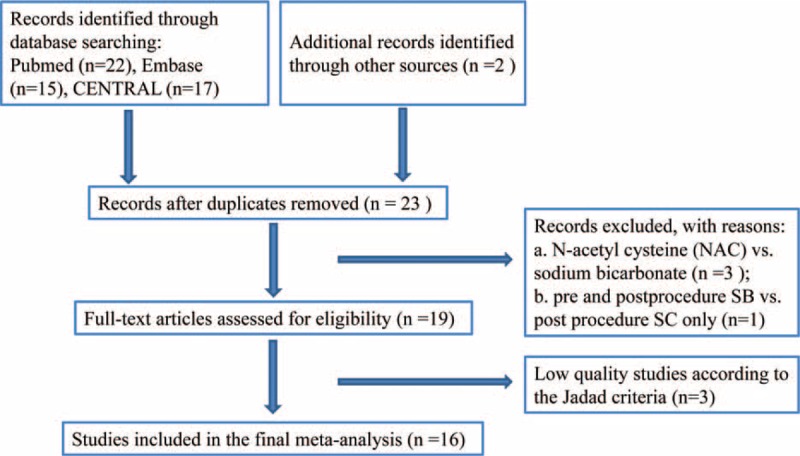
Flow diagram of identification of randomized controlled trials for inclusion.

Literature search retrieved 54 studies from the screened databases of PubMed, EMBASE and CENTRAL and 2 additional records^15,32^ were identified through hand searching. Twenty-three were first removed due to duplication. After initial review of the titles and abstracts of the left 33 studies, 3 studies ^33–35^ comparing N-acetylcysteine (NAC) versus sodium bicarbonate and 1 study regarding hydration with SB pre- and postprocedurally while hydration with SC only postprocedurally were excluded. The remaining 19 studies were further screened into the full texts and 3 studies^36–38^ were rejected because of low quality according to the Jadad score. Finally, 16 studies with 3537 patients were included in our meta-analysis, among which 1768 patients were randomized to the SB group, and 1769 patients were randomized to SC group. The flow chart of the literature search is shown in Figure 1.

### Study Characteristics and Endpoint Measurement

Detailed description of the baseline characteristics of studies included is listed in Additional file 1. A total of 16 studies were included in this meta-analysis, Jadad scores ranged from 3 to 5, only 5 studies had allocation concealment appropriately described^9,16,18,32,39^ while the remaining 11 were unclear. Each of 16 studies involved a sample size ranged from 59 to 502. All of the patients underwent coronary angiography and/or percutaneous coronary intervention with low or iso-osmolar contrast media administrated. Most of the regiments were described as infusion of 154 mEq/L SB or SC at the rate of 1 to 3 mL/kg for 1 to 3 hours before, at the rate of 1 mL/kg during and at the rate of 1 mL/kg for 3 to 6 hours after the procedure. The 2 groups were comparable in age and sex.

### Primary Endpoints

#### Contrast-Induced Acute Kidney Injury

**FIGURE 2:**
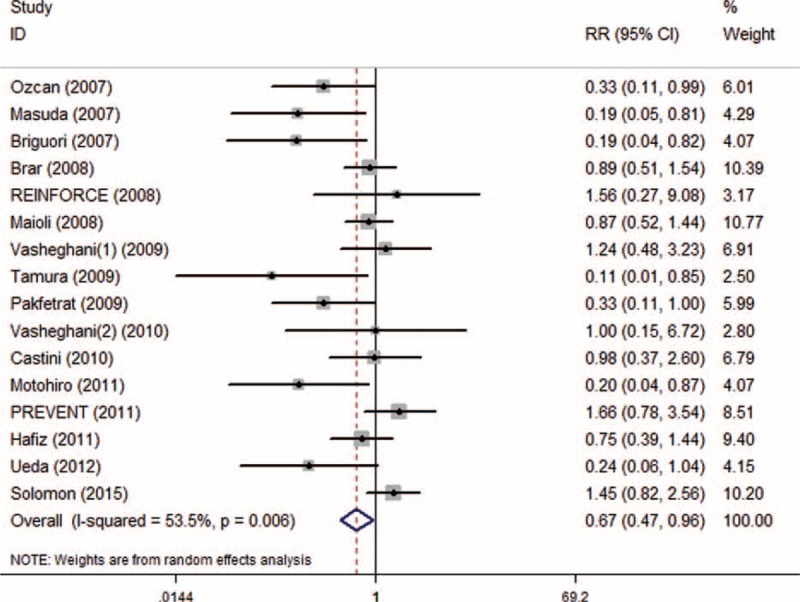
Forest plot of relative risk (RR) for incidence of CI-AKI.

With 16 trials and 3537 patients, 1768 of the total were allocated to SB, and 1769 were allocated to SC group. The overall incidence of CI-AKI was 9.36% ranging from 3.45% to 20.34%. CI-AKI occurred 7.98% in SB group and 10.74% in SC group. Pooled treatment effect analysis revealed that SB versus SC significantly reduced the incidence of CI-AKI (RR 0.67; 95% CI: 0.47 to 0.96, *P* = 0.029), using a random effects model. But significant heterogeneity was observed among the RCTs (*I*^2^ = 53.5%, *P* = 0.006) (Figure 2).

**FIGURE 3:**
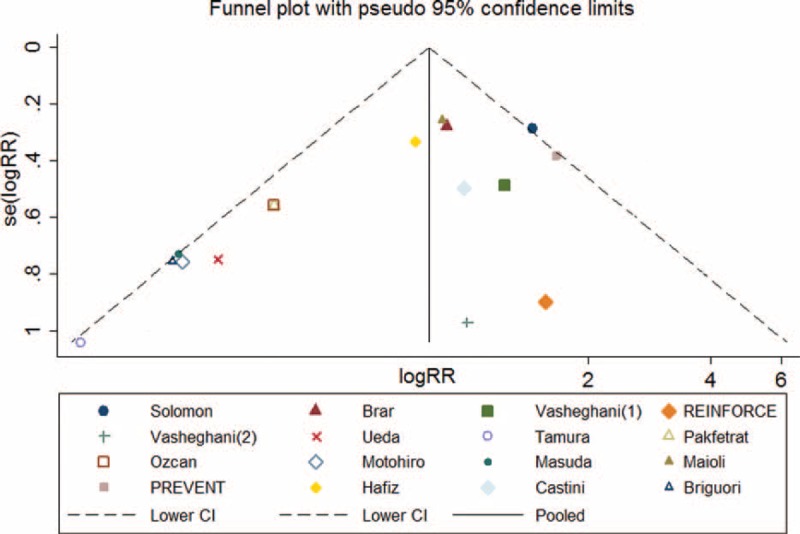
Funnel plot of CI-AKI with pseudo 95% confidence limits.

Asymmetry was observed upon visual inspection of funnel plot. Begg test and Egger test showed that there was potential publication bias among the included RCTs (Begg test, *P* = 0.034; Egger test, *P* = 0.011) (Figure 3).

Meta-regression indicated that sample size was the only source of heterogeneity (Tau^2^ = 0.15, adjusted *R*-squared = 61.25%, *P* = 0.019).

**FIGURE 4:**
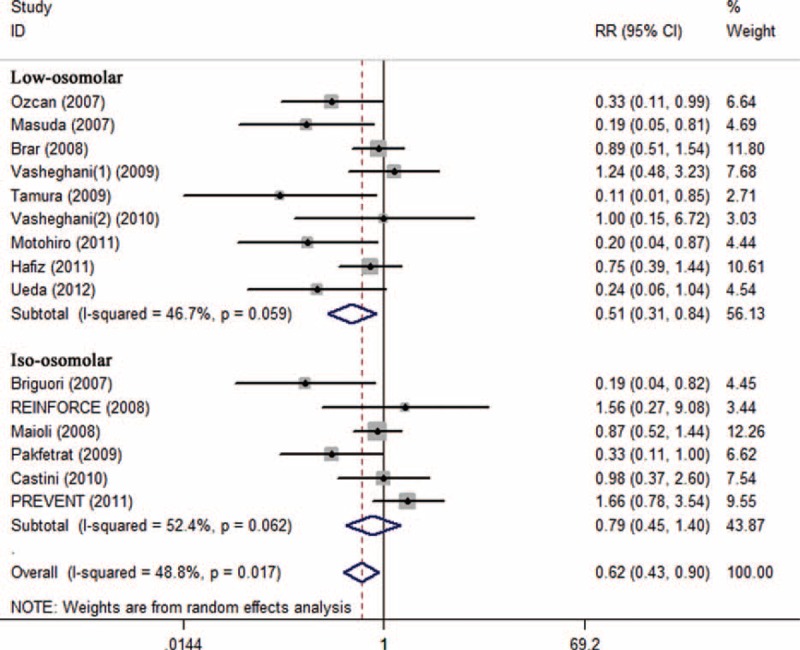
Subgroup analysis of iso-osmolar vs low osmolar contrast media.

**FIGURE 5:**
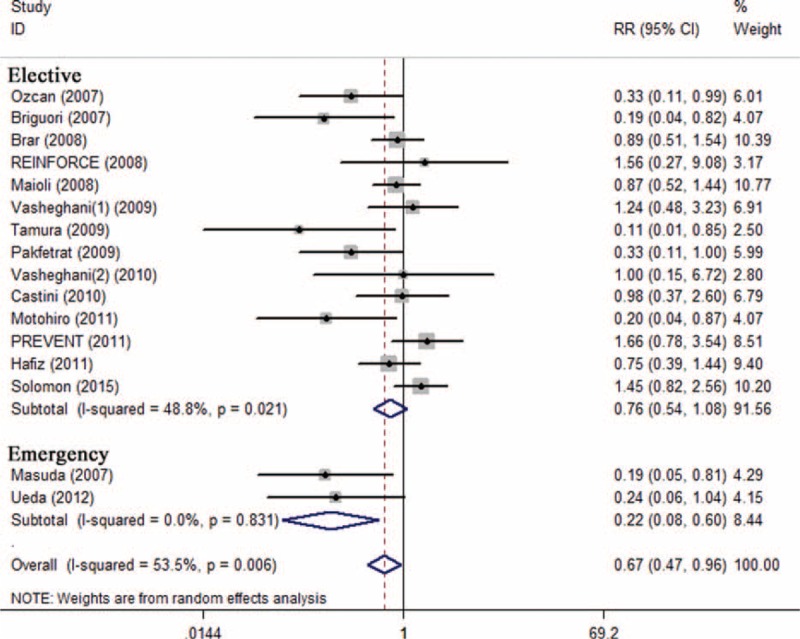
Subgroup analysis of emergency procedures vs elective procedure.

**FIGURE 6:**
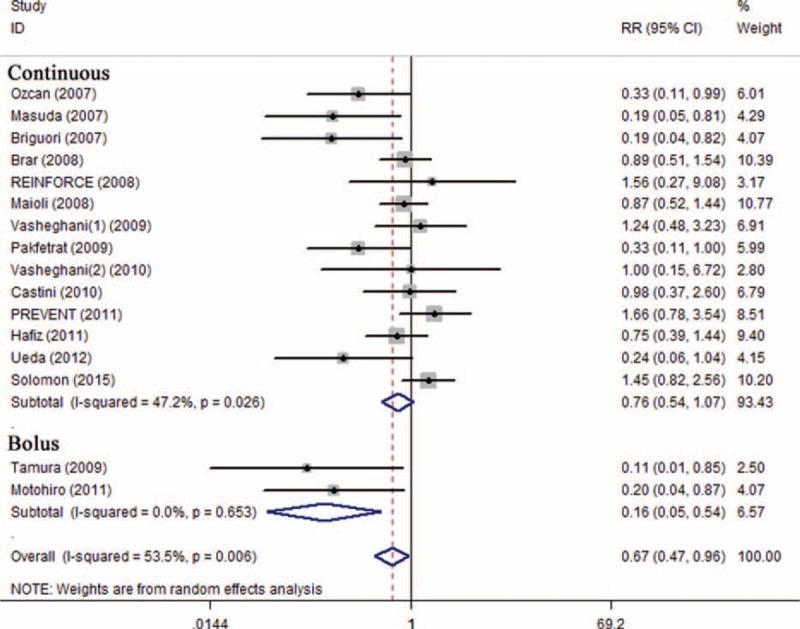
Subgroup analysis of bolus injection vs continuous injection.

**FIGURE 7:**
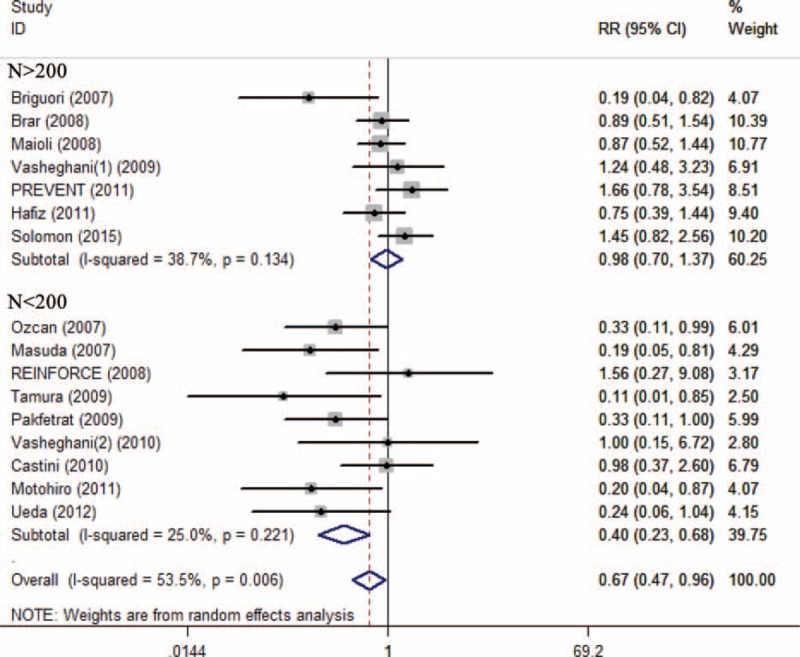
Subgroup analysis of sample size SS >200 vs SS <200.

Subgroup analysis showed that preventive effects of SB were superior to SC on CI-AKI in patients injected with low-osmolar contrast media (n = 1603, RR 0.51; 95% CI: 0.31–0.84; *I*^2^ = 46.7%, *P* = 0.059) compared with iso-osmolar contrast media (n = 1543, RR 0.79; 95% CI: 0.45–1.40; *I*^2^ = 52.4%, *P* = 0.062) (Figure 4), in patients undergoing emergency procedures (n = 118, RR 0.22; 95% CI: 0.08–0.60; *I*^2^ = 0.0%, *P* = 0.831) compared with elective procedures (n = 3419, RR 0.76, 95% CI: 0.54–1.08; *I*^2^ = 48.8%, *P* = 0.021) (Figure 5), and in patients receiving bolus injection (n = 299, RR 0.16, 95% CI: 0.05–0.54; *I*^2^ = 0.0%, *P* = 0.653) compared with continuous injection (n = 3238, RR 0.76; 95% CI: 0.54–1.07; *I*^2^ = 47.2%, *P* = 0.026) (Figure 6). Subgroup analysis by sample size (SS) indicated that the preventive effect of SB in studies with SS less than 200 (n = 1105, RR 0.40; 95% CI: 0.23–0.68; *I*^2^ = 25.0%, *P* = 0.221) was greater than those studies with SS more than 200 (n = 2432, RR 0.98, 95% CI: 0.70–1.37; *I*^2^ = 38.7%, *P* = 0.134) (Figure 7).

**FIGURE 8:**
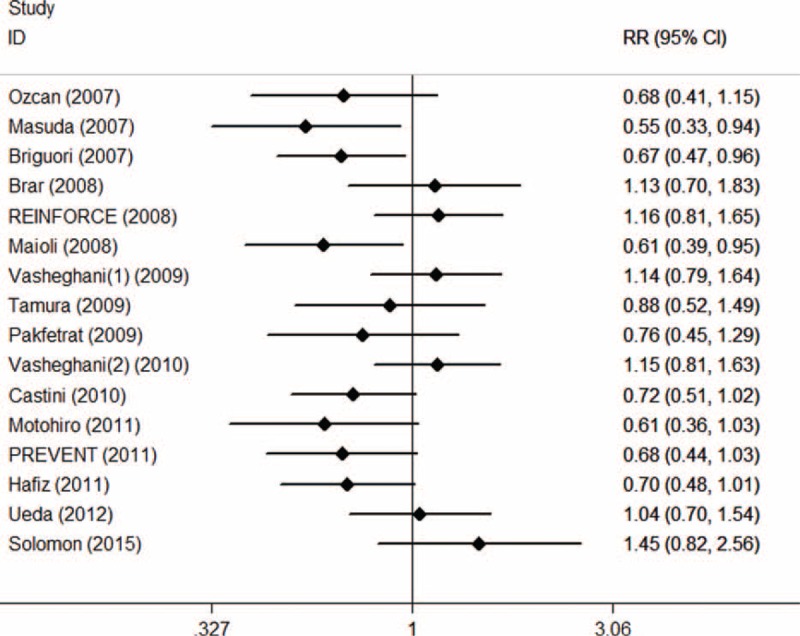
Cumulative meta-analysis plot of incidence of CI-AKI.

Cumulative meta-analysis showed a significant instability in pooled RRs of each year and corresponding 95% CIs did not become narrower with the studies added one by one, indicating that the current studies were not powered enough to lead to a safe conclusion on the significance of the SB hydration toward CI-AKI incidence (Figure 8).

**FIGURE 9:**
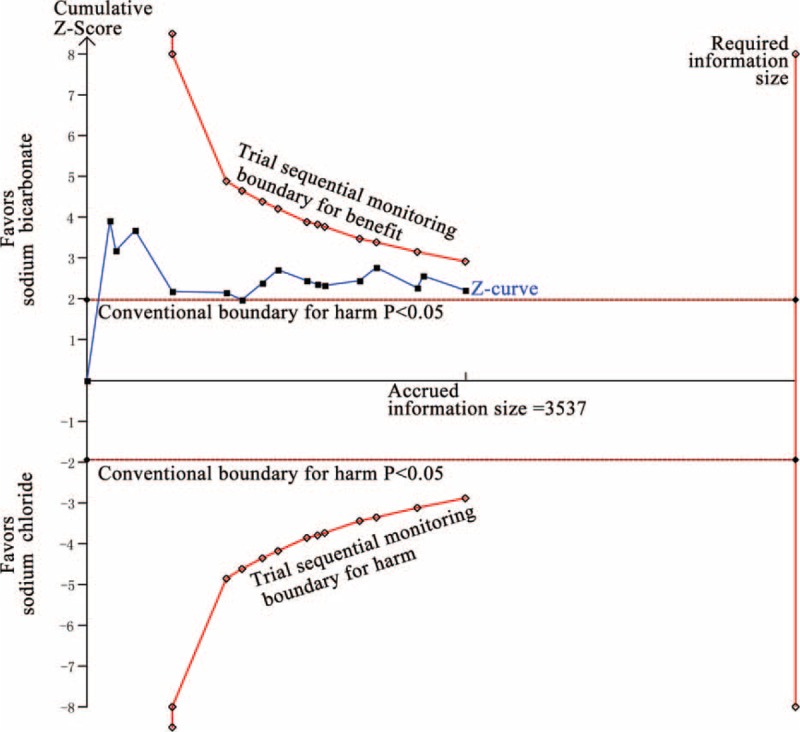
Trial sequential analysis of 16 trials reporting incidence of CI-AKI. The required information size (RIS) of 6614 is based on an anticipated intervention effect of 30% relative risk reduction, a control event proportion estimated from the cumulated incidence of CI-AKI in the sodium chloride arm, and a diversity of 62%, α of 5%, power of 80%. RIS is far from reached and the z-curve crossed only the conventional boundary (*P* < 0.05) without crossing the trial sequential monitoring boundary for benefit, indicating that more trials are needed to build a firm evidence. The trial sequential analysis adjusted 95% confidence interval for a relative risk of 0.67 is 0.47 to 0.96.

In the TSA, the required information size (RIS = 6614) was not reached. The cumulative z-curve crossed the traditional boundary (*P* = 0.05) but failed to cross the sequential monitoring boundary, indicating lack of firm evidence for a 30% reduction in incidence of CI-AKI with SB hydration versus SC hydration (Figure 9).

#### Requirement of Dialysis

**FIGURE 10:**
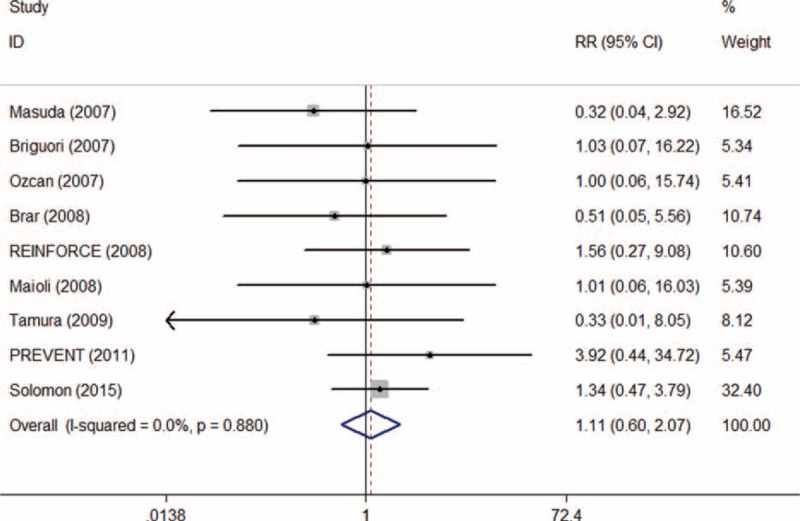
Forest plot of relative risk (RR) for the requirement of dialysis.

A total of 2880 patients were included from 13 RCTs reporting data on requirement of hemodialysis. After removing RCTs with no events in 2 arms, 2371 patients from 9 trials were enrolled in meta-analysis. Of these, 1182 patients were allocated to SB and 1189 were allocated to SC group. The overall rate of requirement of dialysis was 1.60% ranging from 0.39% to 6.78%. Requirement of dialysis occurred 1.69% in SB group, and 1.51% in SC group. Pooled treatment effect analysis revealed that SB versus SC showed no significance in reducing the requirement of hemodialysis (RR 1.11; 95% CI: 0.60–2.07, *P* = 0.729). No significant heterogeneity was observed among the RCTs (*I*^2^ = 0.0%, *P* = 0.880) (Figure 10). At this point, we also conducted correcting on the zero-event trial reporting requirement of dialysis by adding 0.5 to each cell of the 2 × 2 table for the trial and the same result was indicated (RR 1.10; 95% CI: 0.61–1.99, *P* = 0.752) without significant heterogeneity (*I*^2^ = 0.0%, *P* = 0.988).^40^

**FIGURE 11:**
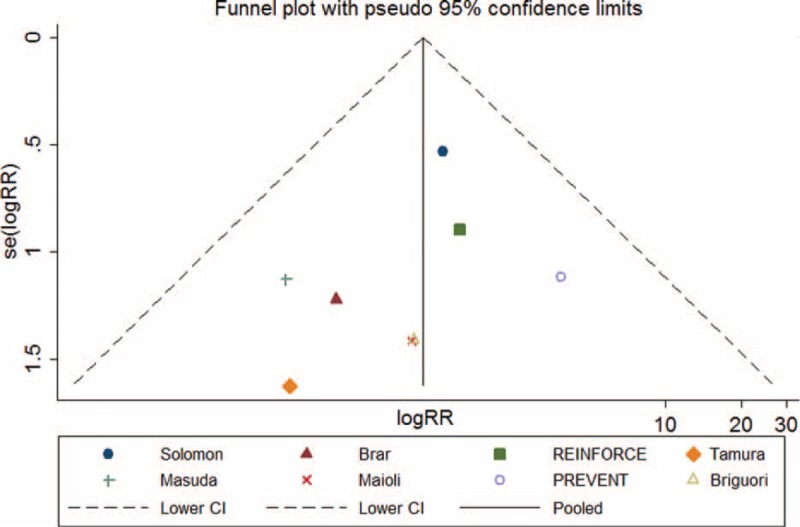
Funnel plot of requirement of dialysis with pseudo 95% confidence limits.

Symmetric was observed upon visual inspection of funnel plot. Begg and Egger tests showed that there was no potential publication bias among the included RCTs (Begg test, *P* = 0.174; Egger test, *P* = 0.334) (Figure 11).

**FIGURE 12:**
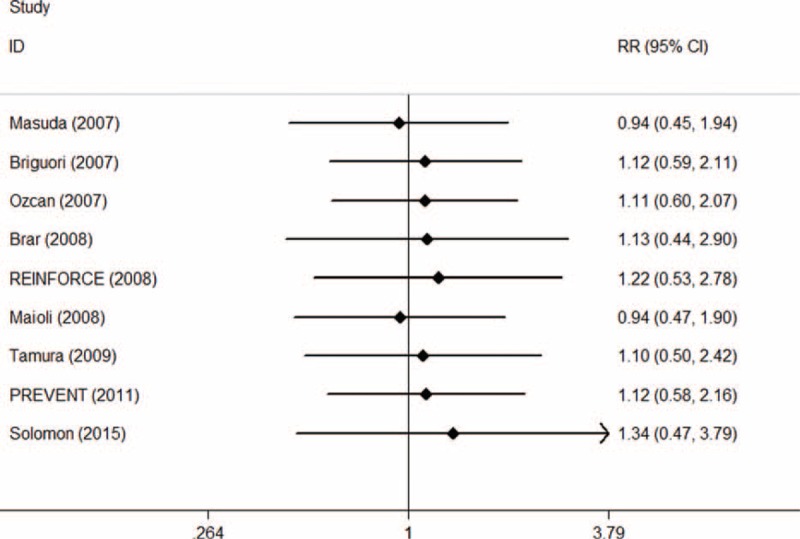
Cumulative meta-analysis plot of the requirement of dialysis.

Cumulative meta-analysis showed a significant instability in pooled RRs of each year and corresponding 95% CIs did not become narrower with the studies added one by one, indicating that the current studies were not powered enough to lead to a firm conclusion on the increase of requirement of dialysis with SB hydration (Figure 12).

**FIGURE 13:**
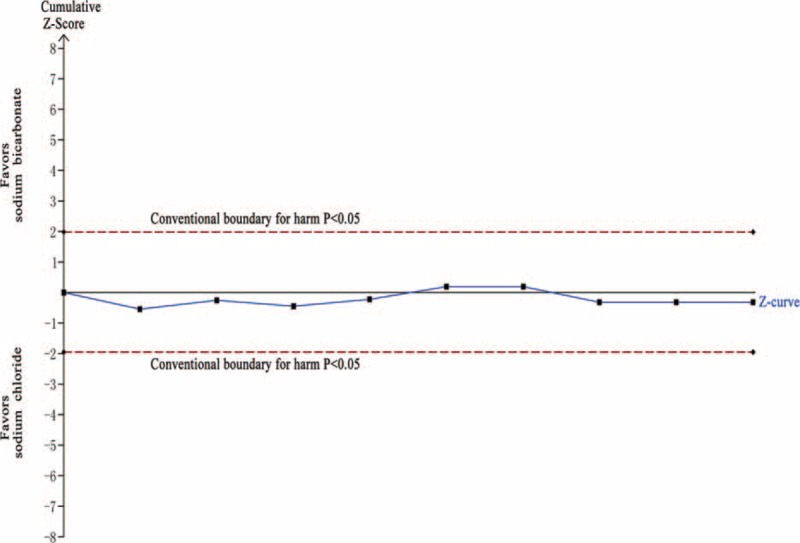
Trial sequential analysis of 9 trials reporting requirement of dialysis. The required information size (RIS) of 170,510 is based on an anticipated intervention effect of 11% relative risk increase, a control event proportion estimated from the cumulated incidence of requirement of dialysis in the sodium chloride arm, and a diversity of 0%, α of 5%, power of 80%. RIS is far from reached and none of the boundaries for benefit or harm has been crossed, leaving the meta-analysis inconclusive of an 11% relative risk increase. The trial sequential analysis adjusted 95% confidence interval for a relative risk of 1.10 is 0.61 to 1.99.

In the TSA, the required information size was not reached (n = 170,510). The cumulative Z-curve crossed neither the traditional boundary (*P* = 0.05) nor the sequential monitoring boundary, indicating lack of firm evidence for an 11% increase in the requirement of dialysis with SB hydration versus SC hydration (Figure 13).

#### Mortality

**FIGURE 14:**
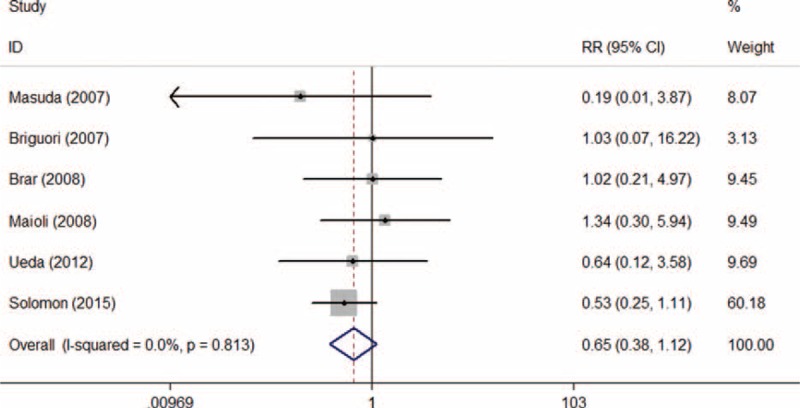
Forest plot of relative risk (RR) for hydration with sodium bicarbonate on mortality.

A total of 2656 patients were included from 11 RCTs reporting data on mortality. After removing RCTs with no events in 2 arms, 1583 patients from 6 trials were enrolled in meta-analysis. Of these, 788 of the total were allocated to SB and 795 were allocated to SC group. The overall mortality was 3.22% ranging from 0.91% to 8.47%. Mortality occurred 2.64% in SB group and 3.79% in SC group. Pooled treatment effect analysis demonstrated that there was no significant beneficial effect for SB hydration to reduce mortality (RR 0.71; 95% CI: 0.41–1.21, *P* = 0.204). No heterogeneity was observed among the RCTs (*I*^2^ = 0.0%, *P* = 0.704) (Figure 14). After trials for zero-event correction, the result was still consistent with the result shown above (RR 0.68; 95% CI: 0.40–1.14, *P* = 0.140) without heterogeneity observed (*I*^2^ = 0.0%, *P* = 0.992).

**FIGURE 15:**
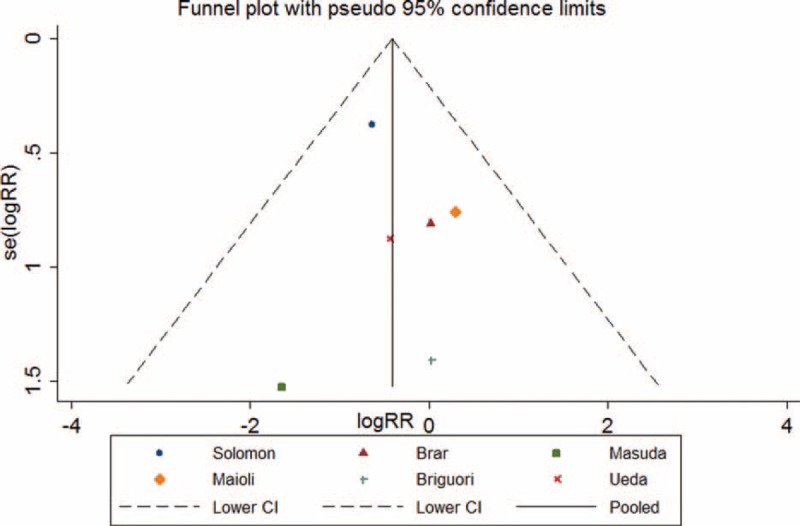
Funnel plot with pseudo 95% confidence limits for hydration with sodium bicarbonate on mortality.

Asymmetric was observed upon visual inspection of funnel plot. However, Begg test and Egger test showed that there was no potential publication bias among the included RCTs (Begg test, *P* = 0.707; Egger test, *P* = 0.521) (Figure 15).

**FIGURE 16:**
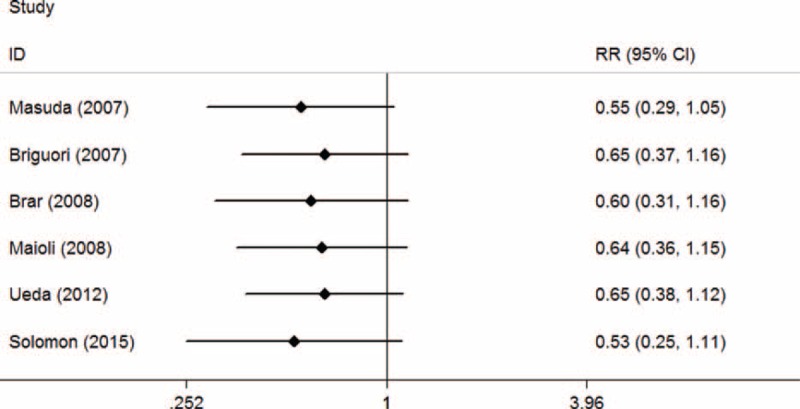
Cumulative meta-analysis plot of mortality. .

Cumulative meta-analysis showed a instability in pooled RRs of each year and corresponding 95% CIs did not become narrower with the studies added one by one, indicating that the current studies were not powered enough to lead to a safe conclusion on the significance of the SB hydration toward mortality (Figure 16).

**FIGURE 17:**
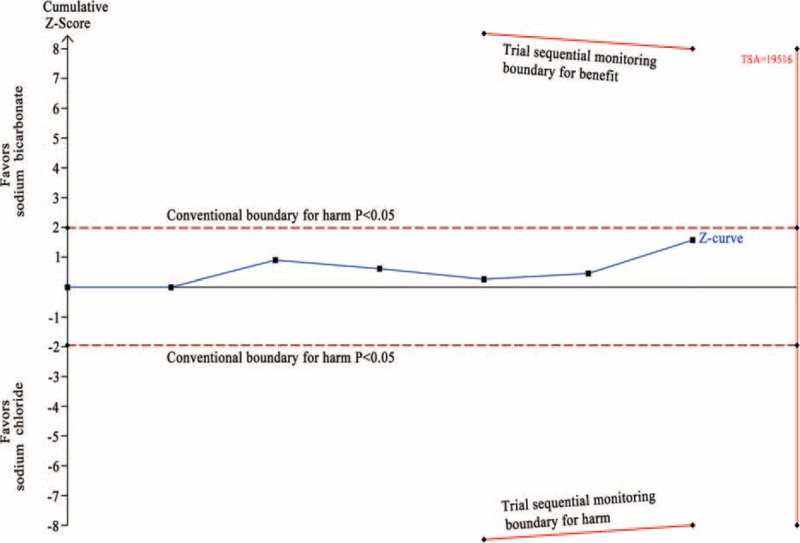
Trial sequential analysis of 6 trials reporting mortality. The required information size (RIS) of 19,516 is based on an anticipated intervention effect of 30% relative risk reduction, a control event proportion estimated from the cumulated mortality in the sodium chloride arm, and a diversity of 0%, α of 5%, power of 80%. RIS is far from reached and none of the boundaries for benefit or harm has been crossed, leaving the meta-analysis inconclusive of a 30% relative risk reduction. The trial sequential analysis adjusted 95% confidence interval for a relative risk of 0.68 is 0.40 to 1.14.

In the TSA, the required information size (n = 19,516) was not reached. The cumulative Z-curve crossed neither the traditional boundary (*P* = 0.05) nor the sequential monitoring boundary, indicating lack of firm evidence for a 30% reduction in the mortality with SB hydration versus SC hydration (Figure 17).

### Secondary Endpoints

#### Length of Hospital Stay

From 396 patients, 201 were allocated to SB and 195 were allocated to SC group. Pooled analysis showed that SB versus SC was not persuasive enough in reducing the length of hospital stay with SMD of −1.47 (95% CI: −4.14 to 1.20; *P* = 0.279) using a random effects model. Significant heterogeneity was observed among the RCTs (*I*^2^ = 69.7%, *P* = 0.037).

#### Change in Serum Creatinine

From 1596 patients, 801 were allocated to SB and 795 were allocated to SC group. Pooled analysis showed that SB versus SC could significantly reduce the change in serum creatinine with SMD of −0.33 (95% CI: −0.55 to −0.12; *P* = 0.003) using a random model. Significant heterogeneity was observed across the RCTs (*I*^2^ = 75.8%, *P* < 0.001).

#### Change in eGFR

From 873 patients, 439 were allocated to SB and 434 were allocated to SC group. Pooled analysis showed that SB versus SC could significantly reduce the change in eGFR with SWD of −0.17 (95% CI: −0.30 to −0.04; *P* = 0.013) using a fixed effects model. Significant heterogeneity was observed among the RCTs (*I*^2^ = 0.0%, *P* = 0.573).

#### Change in Urine pH

From 831 patients, 417 were allocated to SB and 414 were allocated to SC group. Pooled analysis showed that SB versus SC could significantly increase the urine pH with SMD of 0.67 (95% CI: 0.33–1.01; *P* < 0.001) using a random effects model. Significant heterogeneity was observed among the RCTs (*I*^2^ = 82.8%, *P* = 0.001).

## DISCUSSION

### Principal Findings

The result of this meta-analysis with data derived from randomized trials showed a significant reduction in incidence of CI-AKI, the change in SCr, and eGFR as well as an increase in urine pH pre- and postprocedure. However, no significant differences were observed in the reduction of length of hospital stay, requirement for dialysis, and mortality with sodium bicarbonate.

In our current analysis of data from RCTs, one might be tempted to conclude that preparatory hydration with sodium bicarbonate in patients undergoing CAG and/or PCI is the way to give significant benefits, including reduction in incidence of CI-AKI. However, given the total sample size of 3537 patients only, trial sequential analyses showed that the current body of evidence failed to provide a firm evidence for a 30% relative risk reduction in incidence of CI-AKI as well as mortality and a 12% relative risk increase in the requirement of dialysis. The interpretation of the TSA is similar to interim analysis of clinical trials. Here, the interim analysis is performed with every published trial and can dictate whether a sufficient level of evidence has been reached (by the cumulative z-curve crossing the trials sequential monitoring boundary) or the futility boundary is reached. Therefore, in the light of reduction of CI-AKI incidence for patients engaged in preparatory hydration with sodium bicarbonate in PCI and/or CAG procedure, there is still lack of firm evidence to allow definitive conclusion to be drawn.

### Relation to Previous Studies

Hydration to prevent CI-AKI in patients undergoing PCI and/or CAG is very essential in the clinic as it is reported that the incidence of CI-AKI is significantly higher in patients presenting with multiple cardiovascular risk factors, with an incidence as high as 50%.^41^ In 2004, Merten et al^42^ first reported an important reduction in the risk of CI-AKI in patients hydrated with sodium bicarbonate versus sodium chloride (1.7% vs 13.6%, *P* = 0.02).

However, there is no lack of RCTs leading to the contrary conclusion. In 2012, Gome et al conducted an RCT of 302 patients undergoing PCI and CAG showed that sodium bicarbonate was not superior to saline for prevention of CI-AKI with the incidence of CI-AKI (6.1%) in the sodium bicarbonate group and (6.0%) in the saline group (*P* = 1.0), the changes in serum creatinine (0.01 ± 0.26 mg/dL vs 0.01 ± 0.35 mg/dL, *P* = 0.9), and eGFR (0.9 ± 8.0 mL/min vs 2.3 ± 10 mL/min, *P* = 0.2).^29^

As far as we are concerned, there are a few meta-analyses about the preparatory hydration of patients undergoing PCI and CAG specifically to prevent CI-AKI. A meta-analysis was published in 2015 including 5698 patients from 28 trials assessing the benefits of sodium bicarbonate in coronary angiography. Results indicated reduction in the incidence of CI-AKI (OR 0.718, 95% CI 0.60–0.85), but no significant reductions in mortality (RR 0.73, 95% CI: 0.42–1.26), rate of dialysis requirement (RR 1.00, 95% CI: 0.49–2.01) compared with sodium chloride,^29^ which were in line with our findings. But it was a meta-analysis of 28 RCTs that involved 5698 patients who underwent many types of procedures with radiological contrast and not only PCI or CAG, which means that the administration route of the contrast agent was not intra-arterial. Like most of the meta-analyses, this study also performed cumulative analyses; however, cumulative analyses are at risk of producing random errors because of repetitive testing on accumulating data.^43^ Therefore, false-positive or negative conclusion could be drawn in the previous meta-analysis without a trial sequential analysis.^44^

From our meta-analysis including 3537 patients and correcting for zero-event trials, we did find a significant reduction in the incidence of CI-AKI with sodium bicarbonate. As was shown on trial sequential analysis, the total number of patients was still too low to reach firm conclusions, and from the RR reduction found in the previous meta-analysis, an information size of 6614 patients is needed and at the moment only 54.48% of this number has been randomized. In the light of reduction in requirement of dialysis and mortality, no significance had been found in our meta-analysis. On the basis of TSA, an information size of 170,510 and 19,516 patients are needed and at the moment only 1.69% and 13.61% of these numbers have been randomized respectively for dialysis and mortality, indicating that more randomized trails are needed to allow firm conclusions to be drawn.

### Strengths and Limitations

Our meta-analysis offers a number of strengths. First, as far as we were concerned, very few randomized trials comparing sodium bicarbonate versus sodium chloride were conducted with high methodological quality, which gives ample room for systematic errors (bias). Second, we excluded those studies with low quality (Jadad scale was less than 2) to guarantee a high quality of RCTs. Third, apart from the quality assessment, the methodological analysis is thorough and comprehensive to minimize the risk of systemic errors. Total methods included heterogeneity analysis, meta-regression, sensitivity analysis, publication bias analysis, subgroup analysis, cumulative meta-analysis. Finally, we conducted trial sequential analyses, which we consider a useful instrument to evaluate when firm evidence may be reached.

There are some limitations in our meta-analysis, including the restriction on quality and quantity of available evidence, the variability in inclusion and exclusion criteria among the studies, and variability in the follow-up duration. Apart from the above, the included patient population in randomized trials may not be representative for a general patient population. Another aspect that our and other meta-analyses are encumbered with is that the heterogeneity for half of the outcomes is significant, suggesting that the result might identify with the irrespective of the speculations mentioned above and the authors were failed to contact for the missing information.

### Implications

Even after the 16 RCTs with 3537 patients included, we are not sure if sodium bicarbonate is better than sodium chloride in reducing the incidence of CI-AKI for patients undergoing PCI and/or CAG. Our meta-analysis has to be updated when results from these trials are available to see if the firm conclusion could be drawn or not.

To increase the strength of evidence on which method of preparatory hydration to prefer in patients undergoing PCI and/or CAG to reduce the incidence of CI-AKI and other relevant outcomes, we recommend inclusion of larger and higher quality of randomized trials with more strict conduction in blinding, allocation concealment and longer follow-up, etc.

Hydration with sodium bicarbonate could protect renal function by reducing the change in SCr and eGFR pre- and postprocedurally as well as by increasing urine pH as SB is not only related to the higher volume expansion and but also acts as a alkalizational scavenger for peroxynitrite and other reactive species generated from NO (Fenton reaction).^45^

## CONCLUSIONS

Data from randomized trials indicated significant reduction in incidence of CI-AKI, the change in serum creatinine, and the change in estimated glomerular filtration rate as well as an increase in urine pH but no significant differences were observed in the reduction of length of hospital stay, requirement for dialysis and mortality with sodium bicarbonate versus sodium chloride for patients undergoing PCI and/or CAG. However, with a total of only 3537 patients, our analyses indicated that there is no firm evidence to support reduction in incidence of CI-AKI, requirement of dialysis and mortality with sodium bicarbonate according to the trial sequential analysis. The results of this study lend credence to the continuation of more high-quality RCTs on preparatory hydration with sodium bicarbonate as the evidence on the primary outcomes is inconclusive.

## Supplementary Material

Supplemental Digital Content

## Supplementary Material

Supplemental Digital Content
