# Supplementary material for: How Strong Is the Evidence for Sodium Bicarbonate to Prevent Contrast-Induced Acute Kidney Injury After Coronary Angiography and Percutaneous Coronary Intervention?
Source: Medicine (Baltimore). 2016 Feb 18;95(7):e2715. doi: 10.1097/MD.0000000000002715 (PMC4998610; doi:10.1097/MD.0000000000002715)
Supplement: Supplemental Digital Content [file medi-95-e2715-s001.pdf]

Table 1. The baseline characteristics of included studies and patients

Study	Sample size	Age		Male (%)		Contrast media	Definition of CI-AKI	Regiment	Endpoints
		SB	SC	SB	SC				
Solomon et al [32]	391	72±10	72±9	57	58	NA	≥0.5mg/dl or 25% rise in serum creatinine from baseline during the first 3 days	5 ml/g SB over 1h before, 1.5 ml/kg/h during and 4h after the procedure	CIAKI, RRT, eGFR≥2 0%, death
Brar et al [18]	353	71.0	71.0	62	65	low-osmolar	≥25% reduction in eGFR, ≥25% increase of SCr	3 ml/kg SB for 1h before, 1.5 ml/kg/h during and 4h after the procedure	CIN, dialysis, mortality, eGFR, MI
Vasheghani(1) et al [22]	265	62.9±10.0	63.8±9.0	84	82	low-osmolar iohexol (mainly)	≥0.5 mg/dl or ≥25% increase in SCr 48h after contrast exposure	8.4% SB, 3 mL/kg/h for 1h and 1 mg/kg/h for 6h after the procedure	CIN, LHS, urine pH
REINFORCE et al [39]	145	70.1±8.4	72.7±6.6	75	81	iso-osmolar	≥0.5 mg/dl or ≥25% increase in SCr 48 h after contrast exposure	2 ml/kg/h SB for 2 h before, 1 ml/kg/h during and 6h after the procedure	CIN
Vasheghani(2) et al [16]	72	61.4	62.7	78	81	low-osmolar iohexol	≥0.5 mg/dl or relative ≥25% increase in SCr 48 h after contrast exposure	8 .4% SB, 3 ml/kg/h for 1h before, 1 mg/kg/h for 6h after the procedure	CIN, LHS, urine pH
Ueda et al [14]	59	77.0±9.0	75.0±10.0	77	79	low-osmolar	>0.5 mg/dl or>25% increase in SCr within 2 days after contrast exposure	0.5 mg/kg/h SB before, 1 ml/kg/h during and 6 h after the procedure	CIN, LHS, SCr, mortality
Tamura et al [12]	144	72.3±9.9	73.3±7.7	92	83	low-osmolar iohexol	>0.5 mg/dl or>25% increase in SCr within 3 days after contrast exposure	blous 20 ml SB for 5 min and 1 mg/kg/h for 12 h before and after procedure	CIN, SCr, adverse clinical events
Pakfetrat et al [9]	192	57.8±11.2	58.5±11.5	58	65	iso-osmolar	RIFLE criteria	3 ml/kg/h SB 1h before, 1ml/kg/h for 6h after procedure	CIN, SCr, eGFR, renal failure
Motohiro et al [10]	155	71.0±9.0	74.0±7.0	76	64	low-osmolar	≥0.5 mg/dl or relative ≥25% increase in SCr 2 days after contrast exposure	bolus 1ml/kg/h SB for 3h before to 6h after procedure	CIN, SCr, eGFR, urine pH,
Masuda	59	75.0±8.0	76.0±11.0	63	59	low-osmolar	>0.5 mg/dl or >25% increase	3ml/kg/h SB for 1h before, 1	CIN, SCr,

et al [11]							in SCr within 2 days after contrast exposure	mg/kg/h for 6h after the procedure	urine pH, death
Maioli et al [17]	502	74.0	74.0	57	61	iso-osmolar	absolute increase of SCr ≥0.5 mg/dl within 5 days	3 ml/kg/h SB for 1h before, 1 mg/kg/h for 6h after the procedure	CIN, requiring hemodialysis, mortality
PREVENT et al [23]	382	65.8	67.5	71	71	iso-osmolar	>25% or >0.5 mg/dl increase in SCr within 48 h after contrast exposure	3 ml/kg/h SB for 1h before, 1 mg/kg/h during and 6h after the procedure	CIN, requiring hemodialysis, mortality, MI, stroke
Ozcan et al [19]	264	68.0	70.0	76	75	low-osmolar	>25% or 0.5 mg/dl increase in SCr after 48 h.	1 ml/kg/h SB for 6h before, 1 ml/kg/h for 6 h after the procedure	CIN, BUN, SCr, creatinine clearance
Hafiz et al [13]	320	74.0	73.0	57	57	low-osmolar iodixanol, etc.	≥0.5 mg/dl or ≥25% increase in SCr 2 days after contrast exposure	3 ml/kg/h SB for 1h before, 1 ml/kg/h for 6 h after procedure	CI-AKI, cardiovascular events, death
Castini et al [8]	103	70±8.3	72.7±8.2	85	84	iso-osmolar iodixanol	≥25% or ≥0.5 mg/dL increase in SCr	1 ml/kg SB for 12 h before and 12 h after contrast injection	CIN, SCr
Briguori et al [15]	219	70±9.0	71±9.0	88	81	iso-osmolar	increase in SCr ≥25% over the baseline 48 h after the procedure	3 ml/kg/h SB for 1h before, 1 ml/kg/h during and 6 h after the procedure	CIN, dialysis, SCr, eGFR, dialysis

Note: NA: not available

CI-AKI: contrast-induced acute kidney injury

RRT: renal replacement therapy MI: myocardiac infarction

LHS: length of hospital stay

SCr: serum creatinine

Adverse clinical events: includes pulmonary edema, acute renal failure, requiring dialysis, hemofiltration and death

RIFLE[36]: acronym indicating risk of renal failure, injury to the kidney, failure of kidney function, loss of kidney function, and end-stage renal disease

PRISMA 2009 Checklist

Section/topic	#	Checklist item	Reported on page #
**TITLE**			
Title	1	Identify the report as a systematic review, meta-analysis, or both.	1
**ABSTRACT**			
Structured summary	2	Provide a structured summary including, as applicable: background; objectives; data sources; study eligibility criteria, participants, and interventions; study appraisal and synthesis methods; results; limitations; conclusions and implications of key findings; systematic review registration number.	2-3
**INTRODUCTION**			
Rationale	3	Describe the rationale for the review in the context of what is already known.	3
Objectives	4	Provide an explicit statement of questions being addressed with reference to participants, interventions, comparisons, outcomes, and study design (PICOS).	3-4
**METHODS**			
Protocol and registration	5	Indicate if a review protocol exists, if and where it can be accessed (e.g., Web address), and, if available, provide registration information including registration number.	5
Eligibility criteria	6	Specify study characteristics (e.g., PICOS, length of follow-up) and report characteristics (e.g., years considered, language, publication status) used as criteria for eligibility, giving rationale.	5
Information sources	7	Describe all information sources (e.g., databases with dates of coverage, contact with study authors to identify additional studies) in the search and date last searched.	5
Search	8	Present full electronic search strategy for at least one database, including any limits used, such that it could be repeated.	5-6
Study selection	9	State the process for selecting studies (i.e., screening, eligibility, included in systematic review, and, if applicable, included in the meta-analysis).	5
Data collection process	10	Describe method of data extraction from reports (e.g., piloted forms, independently, in duplicate) and any processes for obtaining and confirming data from investigators.	9
Data items	11	List and define all variables for which data were sought (e.g., PICOS, funding sources) and any assumptions and simplifications made.	5-6
Risk of bias in individual studies	12	Describe methods used for assessing risk of bias of individual studies (including specification of whether this was done at the study or outcome level), and how this information is to be used in any data synthesis.	7
Summary measures	13	State the principal summary measures (e.g., risk ratio, difference in means).	7-8
Synthesis of results	14	Describe the methods of handling data and combining results of studies, if done, including measures of consistency (e.g., I^2) for each meta-analysis.	9

PRISMA 2009 Checklist

Section/topic	#	Checklist item	Reported on page #
Risk of bias across studies	15	Specify any assessment of risk of bias that may affect the cumulative evidence (e.g., publication bias, selective reporting within studies).	7
Additional analyses	16	Describe methods of additional analyses (e.g., sensitivity or subgroup analyses, meta-regression), if done, indicating which were pre-specified.	6-9
**RESULTS**			
Study selection	17	Give numbers of studies screened, assessed for eligibility, and included in the review, with reasons for exclusions at each stage, ideally with a flow diagram.	Figure1
Study characteristics	18	For each study, present characteristics for which data were extracted (e.g., study size, PICOS, follow-up period) and provide the citations.	28 Table1
Risk of bias within studies	19	Present data on risk of bias of each study and, if available, any outcome level assessment (see item 12).	8
Results of individual studies	20	For all outcomes considered (benefits or harms), present, for each study: (a) simple summary data for each intervention group (b) effect estimates and confidence intervals, ideally with a forest plot.	Figures 2/10/14
Synthesis of results	21	Present results of each meta-analysis done, including confidence intervals and measures of consistency.	Figures 2/10/14
Risk of bias across studies	22	Present results of any assessment of risk of bias across studies (see Item 15).	Figures 3/11/15
Additional analysis	23	Give results of additional analyses, if done (e.g., sensitivity or subgroup analyses, meta-regression [see Item 16]).	Figures 4/5/6/7/8/ 9/12/13/16 /17
**DISCUSSION**			
Summary of evidence	24	Summarize the main findings including the strength of evidence for each main outcome; consider their relevance to key groups (e.g., healthcare providers, users, and policy makers).	14-16
Limitations	25	Discuss limitations at study and outcome level (e.g., risk of bias), and at review-level (e.g., incomplete retrieval of identified research, reporting bias).	17
Conclusions	26	Provide a general interpretation of the results in the context of other evidence, and implications for future research.	19
**FUNDING**			
Funding	27	Describe sources of funding for the systematic review and other support (e.g., supply of data); role of funders for the systematic review.	19

From: Moher D, Liberati A, Tetzlaff J, Altman DG, The PRISMA Group (2009). Preferred Reporting Items for Systematic Reviews and Meta-Analyses: The PRISMA Statement. PLoS Med 6(6): e1000097. doi:10.1371/journal.pmed1000097

For more information, visit: **www.prisma-statement.org**.
